# This is not a pipe – But how harmful is electronic cigarette smoke

**DOI:** 10.1016/j.bj.2021.05.006

**Published:** 2021-06-04

**Authors:** Sophia Julia Häfner

**Affiliations:** University of Copenhagen, BRIC Biotech Research & Innovation Centre, Lund Group, 2200 Copenhagen, Denmark

**Keywords:** Electronic cigarettes, SARS-CoV-2 mutations, Cancer immunotherapy, G6PD, Epstein–Barr virus vaccine

## Abstract

This issue of the *Biomedical Journal* tells us about the risks of electronic cigarette smoking, variations of SARS-CoV-2 and ACE2, and how COVID-19 affects the gastrointestinal system. Moreover, we learn that cancer immunotherapy seems to work well in elderly patients, how thyroid hormones regulate noncoding RNAs in a liver tumour context, and that G6PD is a double-edged sword of redox signalling. We also discover that *Perilla* leaf extract could inhibit SARS-CoV-2, that artificial neural networks can diagnose COVID-19 patients and predict vaccine epitopes on the Epstein–Barr Virus, and that men and women have differential inflammatory responses to physical effort. Finally, the surgical strategies for drug-resistant epilepsy, computer-supervised double-jaw surgery, dental pulp stem cell motility, and the restitution of the brain blood supply after atherosclerotic stroke are discussed.

## Spotlight on reviews

### This is not a pipe

But only the painting of a pipe, yet a famous one, made in 1929 by the Belgian surrealist painter René Magritte and titled *The Treachery of Images*, nowadays on display in the Los Angeles County Museum of Art.

Smoking underwent one of the most radical turnarounds in terms of reputation during less than the span of a century. The Industrial Revolution of the 19th century allowed for the cheap mass production of cigarettes, very efficient marketing, and the highly addictive properties of nicotine did the rest to tremendously popularise the smoking habit [1]. After an approximate delay of 25 years, an epidemic of lung cancer, a once extremely rare disease, followed. The incidence of primary lung tumours began to rise during the second half of the 19th century and really took off during the beginning of the 20th. Nowadays, lung cancer is the leading cause for cancer death in the world [2].

The first suggestion of a connection with smoking stems from 1912, but most scientific proof was accumulated in the 1930s and 1940s. Irrefutable evidence for the cigarette-lung cancer connection came from a combination of vast population and cohort studies, animal experimentation, pathological observations, and chemical identification of carcinogens. By 1953, the message had begun to make it through to the general public, but met a formidable counter-attack by the tobacco corporations [1].

Indeed, until the 1960s, it was commonplace to encounter billboards depicting celebrities and their favourite cigarette brand, like Frank Sinatra endorsing the brand Chesterfield for example.^1^ Even more ironically, commercials showed physicians and dentists displaying the white lab-coat of ultimate authority and slogans such as “More Doctors smoke Camels than any other cigarette”,^2^ recommending cigarettes to treat asthma and sore throats, or favour weight loss. Not to mention the emphasis on the sexual attractiveness of smoking, as claimed by Tipalet and their catchphrase “Blow in her face and she'll follow you anywhere”.^3^

As a consequence, despite the official declarations by the cancer authorities of many countries, cigarette consumption kept rising in the USA until the early 1980s.

The tables have significantly turned by now. In 2009, the Parisian public transport provider RATP forbade the poster campaign for the movie *Gainsbourg: A Heroic Life* in their stations and vehicles because the famous French singer was exhaling a wisp of smoke on the picture.^4^

While this verges on a serious attempt to be more Catholic than the Pope, real nicotine marketing has been severely limited in most countries, following notably the WHO Framework Convention on Tobacco Control from 2005. The 168 participant nations engaged themselves to ban tobacco advertising, enforce health warnings on products, and protect people from passive smoking in public areas.^5^

Despite these considerable efforts, tobacco use to date dispatches 6 million people prematurely to the afterlife every year, rendering it the top cause of preventable disease worldwide [2,3], partially due to the quarter of a century delay between smoking and the consequences. Robert N. Proctor, Professor of the History of Science at Stanford University, goes as far as to qualify the cigarette as “the deadliest artefact in the history of human civilisation” and calculates that “the value of a life to a cigarette maker is about US$10 000” [1].

As a matter of fact, up to two million of these yearly deaths alone are caused by lung cancer, and vice versa, according to the American Cancer Society, smoking is responsible for 80% of lung cancer deaths, even more in the case of small cell lung cancer (SCLC).^6^ Another third of the remaining death toll through tobacco is ensured by cardiovascular disease [3,4], followed by chronic obstructive pulmonary disease [5]. On top of this come various morbidities, such as the exacerbation of skin disorders [6], an increased risk for neurodegenerative disease [7], periodontal disease [8], and *in utero* developmental defects of the foetal respiratory apparatus, brain, and cardiovascular system [9,10]. Although tobacco smoking has declined recently in high-income countries, at the global scale, the effect is mitigated. China alone accounts by now for 40% of the worldwide cigarette production and consumption [1,3].

But in 2003, a new player entered the field, triggering another unexpected rebound. The “electronic cigarette” is indeed neither pipe nor cigarette, although it can be sold in the shape of both, but an electronic device allowing for nicotine consumption without tobacco [11]. The idea is actually not that new – in 1965, a contraption producing flavoured steam was patented, but never commercialised due to the popularity of traditional smoking. Almost half a decade later, the concept was re-invented by the Chinese pharmacist Hon Lik as a battery-operated filament that heats a mixture of nicotine and flavouring compounds to generate an aerosol to be inhaled. It fell onto way more fertile ground, especially after entering the US and European markets from 2006 on, where the concept was (not always legally) copied and diversified.

A snowball effect of clever marketing as a safer alternative to conventional smoking, legal loopholes in the restrictions of tobacco use, and a fascinating social phenomenon perhaps fuelled by the increasing social stigma of cigarette smoking, and the strong community feeling generated by virtual platforms ensued [11]. A passionate “vaping community” established a vast online presence and put an impressive amount of time, effort, and even hostility into advertising their enthusiasm for e-cigarettes [12].

Astonishingly similar to the aggressive marketing strategies by the tobacco industry in the 1950s and 1960s, e-cigarettes have been promoted through celebrity-endorsement, emphasising the sexual appeal or targeting the youth, and even questionable scientific support, like the claim “scientists say that electronic cigarettes could save millions of lives”, that spread like a wildfire on popular news websites in 2013.^7^

A series of “e-cigarette or vaping product use-associated lung injury” (EVALI) cases later, warning voices have finally managed to make themselves heard, and the regulations caught up enough to somewhat curtail the surge, but research is only beginning to thoroughly pinpoint and quantify the exact extent of health damage caused by this new toy.

**Fig. 1:**
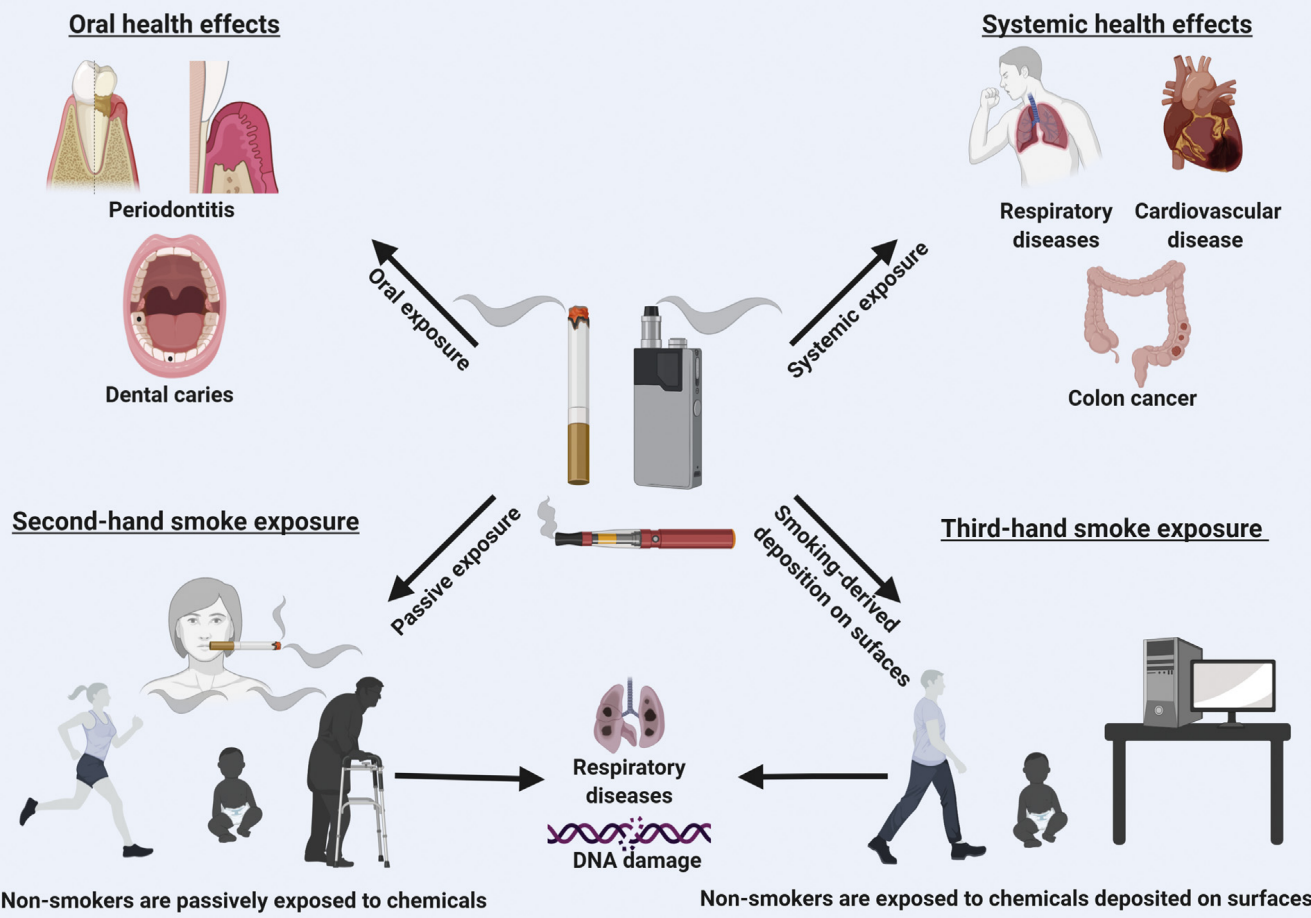
**Direct and indirect impacts on health by smoking.** Examples of the detrimental effects of conventional and electric cigarette smoke on oral and systemic health, and how second or third hand smoke can exert damaging effects. Figure kindly provided by Almeida-da-Silva et al. [13]. See main article for details.

The spotlight of this *Biomedical Journal* issues goes to a highly informative review by Almeida-da-Silva et al., who summarise the current knowledge regarding risk factors and health effects of e-cigarettes compared to traditional cigarettes [13] [Fig. 1].

The authors first remind the reader of the tremendous damage to global heath and economy done by conventional smoking, then provide a short description of the mechanism common to “electronic nicotine delivery systems” (ENDS), as the formal name by the FDA says.

Subsequently, they look into the composition of the liquid inside the devices. Compared to cigarettes, the amount of substances is indeed greatly reduced, and the concentration of some nefarious chemicals reduced. Nevertheless, the aerosol contains nanoparticles, heavy metals, formaldehyde, acetone, and other carcinogenic substances [11]. One obstacle to rigorous studies is the considerable variation in composition and concentration of the “e-juice” between manufacturers. Other components, like those used to generate the countless flavours, have not been studied for their impact on health before. But most importantly, the devices still contain considerable amounts of nicotine, whose deleterious effects on brain development, cognitive function, and the cardiovascular system are beyond debate [14,15]. Some e-cigarettes, such as those of the brand “Juul”, highly popular among young people in the US, even deliver higher nicotine concentrations than conventional smoking [11].

Following, Almeida-da-Silva et al. draw attention to another body part that has very much grown on health experts over the past decades – the microbiome. Without surprises, numerous studies have shown that traditional smoking correlates with modifications to the oral, lung, and gut microbiome, as well as with related diseases such as Crohn's disease and periodontitis [16,17]. Here, the authors focus specifically on the oral microbiome, although the currently available studies have not yet come to a consensus about the effect of e-cigarette use [13].

The strongest alert regarding the dangers of vaping devices came without a doubt in 2019 from a series of cases of severe respiratory problems (EVALI), leading to hospitalisations and deaths [18,19]. As the authors point out, the *corpus delicti* is ironically a vitamin: alpha tocopherol, aka vitamin E, clearly not meant to end up directly in the lung, although other toxic substances are likely to contribute [11,18].

Finally, Almeida-da-Silva et al. question the claim that e-cigarettes could serve as a means to quit smoking, under which they were initially marketed in some countries [11]. They emphasise that the discrepancies between studies are strongly correlated to different levels of restriction-enforcements and public perception of e-cigarette consumption, taking the USA and the UK as examples [13].

Not to mention the real risk of the tobacco industry still exerting an influence on certain studies [20]. Recent proof for this issue has come straight from the COVID-19 pandemic context. Nicotine has been shown to interfere with the renin-angiotensin system, which comprises the angiotensin-converting enzyme 2 (ACE2) receptor, infamous for binding SARS-CoV-2 [21,22]. Although all studies seem to agree that smoking increases the risk for the development of more severe forms of COVID-19 [[23], [24], [25]], an article claiming the opposite just got retracted from the *European Respiratory Journal* for having failed to disclose that the authors had received financial support from the tobacco industry.^8^

In a nutshell, no definitive judgement on e-cigarettes can be rendered yet, at least in terms of a clear comparison to traditional nicotine consumption – the broad diversity of products, user habits, and differences on approaching the regulation between countries impacts on the coherence of studies. Moreover, similar to conventional smoking, many health effects will probably only manifest in a decade or two. It is however by no means a harmless pastime, but rather paves the road to addiction for youth, increases the risk of severe lung injury, and holds potentially other severe, yet unknown side effects in store [11].

One thing is sure, the history of traditional smoking teaches us the weight of popular knowledge and opinion, or the “history of ignorance”, as Proctor calls it [1]. Vaccination has already experienced the prize of social media-exacerbated ignorance in the recent years [26], and any attempt to keep vaping under control has to take into account the power of the internet, the appeal of the forbidden … and the notion of consumer choice and responsibility, for which a certain satirical black comedy from 2005 makes quite a valid point for the latter.

The title? *Thank You for Smoking*.

## Also in this issue

### Reviews

#### High mutation run

In moments of the darkest humour, one might think that we are currently living through a live action role playing session of *Plague Inc.*,^9^ the highly popular video game simulating the spread of a pathogen with the simple goal of eradicating humanity. One crucial gameplay mechanic is the feature “Mutation”, where the plague evolves randomly an ability or symptom, such as “Immune Suppression” or “Extreme zoonosis”. Accurately, the mutation chances are highest if the player selects “virus” for their pathogen of choice.

“Coronavirus” was an astute choice, for sure, given that this type belongs to the most rapidly evolving viruses. By now, the fear of SARS-CoV-2 variants has already become bitter reality, and caused new waves in infection, such as the B.1.627.2 version, which just re-precipitated India into a state of advanced emergency. The biggest – justified - fears associated with the emergence of these variants is that the virus might improve its abilities to escape the immune system and be less responsive to vaccines [27,28].

Huang et al. present a comprehensive review that encompasses the genomic architecture of coronaviruses and the most recent state of knowledge regarding all identified mutations and their impact on viral properties, with a special focus on the interaction between the SARS-CoV-2 spike protein and the hijacked host receptor ACE2. Into the bargain, the authors consider also the numerous genetic variants of the latter, and how these could potentially affect binding and virion intake [29].

#### Local dialects of infection

One characteristic of SARS-CoV-2, which quickly became apparent, is that the virus wreaks havoc not only in the respiratory tract, but also other organs such as the myocardium, digestive system, liver or pancreas [30]. As such, diarrhoea was soon added on the list of potential COVID-19 symptoms [31].

Pola et al. dedicate this brief review to the complex relationship of the virus with the gastrointestinal (GI) tract, the liver, and the pancreas [32]. They begin with a reminder of the viral transmission process and entry into host cells by hijacking the angiotensin-converting enzyme 2 (ACE2) as a receptor and the protease furin for spike (S) protein activation, and provide a concise description of ACE2's physiological roles within a crucial system in charge of controlling blood pressure and electrolytes. Predictably, the involvement of SARS-CoV-2 with many organs is the direct consequence of the widespread ACE2 tissue expression pattern. Subsequently, the authors go over organ-specific roles of ACE2 in the abovementioned organs, as well as how this mediates local infection, damage, and transmission by SARS-CoV-2. Specific attention is paid to the intricate cross talk between the lung, gut, immune system, and microbiome, likely to be perturbed by and potentially amplify COVID-19 disease [32].

#### The old man and the T-cell

SARS-CoV-2 has placed the emphasis on one of the most vulnerable populations in the context of a pandemic, which is the elderly. The risk of developing severe COVID-19 and mortality were directly proportional to age, with individuals over 65 years old considered particularly vulnerable [33]. As vaccination is assumed to be less efficient in the elderly, there were concerns regarding the ongoing vaccination programs, which started in most countries with the oldest citizens [34].

However, this assumption might not be of general truth, as Granier et al. point out in their review dedicated to the immune response of older subjects, in particular to cancer immunotherapy [35]. The latter represents the latest beacon of hope for cancer treatment, to a degree that Honjo and Allison received the Nobel Prize in Physiology and Medicine 2018 for discovering how to release the brakes of the immune system by blocking the T-cell proteins CTLA-4 and PD-1 with antibodies [36]. Granier et al. investigate here how efficient these therapies are in the elderly, which are underrepresented in clinical studies because of frequent comorbidities. The authors provide a very interesting overview on the complex aging process of the immune system, or “immunosenescence”, which does not entirely correlate with decreasing performance of the defences, as well as the currently available data on the safety and clinical response of immunotherapies in older patients. Altogether, their findings are reassuring and point towards a better tolerance and response to the novel treatment strategy than expected, and especially when compared to radio- or chemotherapy [35].

#### Rise of the RNA

The rise of high throughput sequencing technologies shortly after the start of the new millennium, paralleled by the drop of sequencing costs, unveiled a whole brave new word – the one of noncoding RNAs (ncRNAs). Up to this point, the fact that protein-coding genes only make up for 2% of the human DNA had been considered a mere proof that evolution is neither a planned nor a particularly optimised process, given the amounts of “junk DNA” present in every single cell. When it became clear that “coding” is rather the exception than the rule, faced with manifold species of noncoding transcripts arising from the “junk” and their crucial roles in literally every cellular process [37,38], the scientific world witnessed first an explosion of micro-RNAs (miRNAs), followed by a gold rush for long ncRNAs (lncRNAs), peaking somewhere around 2015. Especially their role in disease, first and foremost cancer, has been explored, to a degree that the famous “hallmarks of cancer” were rewritten to include ncRNAS [39].

One can easily loose track in the complex world of ncRNAs, but Huang et al. manage to focus on a concrete subset in their present review, centred on the role of ncRNAs controlled by thyroid hormones in liver cancer [40]. After a helpful introduction to all three abovementioned elements and their connections two by two, the authors provide a concise description of thyroid hormone-controlled miRNAs and lncRNAs with oncogenic or tumour-suppressor roles in liver cancer. In addition, their molecular mechanism as well as potential uses in the form of therapeutic targets or biomarkers are discussed.

#### Doctor redox and mister ROS

Cancer is the master of hijacking, excelling in the expropriation of virtually any cellular process to suit its needs of fast-track cell growth and resistance to hostile environments or treatment. Mastering metabolism and oxidative stress are core competences required to achieve these [41]. The glucose-6-phosphate dehydrogenase (G6PD), a key player of the pentose phosphate pathway, happens to be involved in both. Moreover, the enzyme can assume anti-just as much as pro-oxidative roles, rendering it an ideal candidate for cancer to tip the balance into its favour [42].

Here, Yang et al. describe in detail the multiple pathways and interaction partners through which G6PD promotes the different hallmarks of cancer, and how this qualifies it as an attractive therapeutic target [43].

### Original articles

#### Antiviral mojito

Vaccination programs against SARS-CoV-2 are currently in full swing, but the continuous emergence of variants and uncertain duration of protection by the vaccines keep the search for additional treatment options going.

Concomitantly, drug repurposing attempts and the investigation of natural compounds have the wind in their sails, given that their official approval would be faster than for *de novo* synthesised compounds [44]. Moreover, the use of traditional medicine to treat COVID-19 has been officially encouraged in several Chinese provinces [45].

On this account, Tang et al. screened a selection of herbs from traditional Chinese medicine for the reduction of SARS-CoV-2 induced death in cell cultures [46]. Extracts obtained from *Perilla frutescens* (PLE), a member of the mint family, displayed remarkable abilities to block viral entry as well as RNA and protein synthesis, especially if added early after infection. In addition, PLE inhibited the expression of pro-inflammatory cytokines, the causative agents of the feared cytokine storm characteristic of severe COVID-19. Finally, the substance harboured an additive-to-synergistic effect with remdesivir, the only currently approved antiviral against SARS-CoV-2. If the plant can live up to the engendered hopes in an *in vivo* model, it should be seriously considered for further investigations.

#### A GigaByte of help

Hospitals all over the world were overwhelmed several times with floods of patients during the peaks of COVID-19. Fast and accurate patient classification according to the likelihood of SARS-CoV-2 infection and risk to develop severe COVID-19 depending on comorbidities is instrumental to cope with this type of emergency situations.

Computer-aided diagnosis was suggested as early as 1978 [47], but has really taken off in recent years thanks to the emergence of artificial neural networks (ANN) and machine learning, notably for medical image analysis [48]. COVID-19 has proven to be another important training ground [49].

Here, Mohammadi et al. investigate first the main characteristics, symptoms, and underlying diseases in COVID-19 patients from six Iranian regions, revealing regional differences in the top hits per category [50]. Subsequently, the authors separately train a linear regression model and a neural network to identify true SARS-CoV-2 positive patients based on these datasets. Both models revealed to be highly accurate, with the neural network performing best.

#### Fatal kiss

SARS-CoV-2 has taken centre stage for over a year by now, but countless other viruses are queuing for attention. Infection by the Epstein–Barr virus (EBV) for instance, also known as human gammaherpesvirus 4, has taken place in up to 90% of the adult world population. The virus attacks specifically epithelial and B-cells, and persists in the latter for the host's lifetime.

EBV mainly causes the fairly harmless infectious mononucleosis, also known as “kissing disease”, because the virus is principally transmitted by saliva [51]. However, the pathogen can also trigger the development of certain cancer types, such as several forms of lymphoma, nasopharyngeal carcinoma, or stomach cancer, and might be involved in multiple sclerosis, thus rendering a vaccine highly desirable [52].

Olotu and Saliman here present very promising data regarding the prediction of B- and T-cell epitopes for potent vaccines against EBV [53]. They provide the reader first with a detailed description of the viral life cycle and proteome, along with the different layers of reactions to the virus by the innate and adaptive immune system. Prior attempts to develop vaccines against EBV and their shortcomings, but also the recent successes of peptide-based vaccines against other viral types are briefly reviewed. An exhaustive description of the rigorous identification and validation of B-cell and T-cell epitopes on EBV proteins related to epithelial cell attachment, DNA synthesis and replication, and capsid assembly ensues. Using multiple cutting-edge modelling tools, such as artificial neural networks, the authors assess and simulate the accessibility, antigenicity, and binding dynamics of the candidate sites, providing a cornucopia of material to be hopefully further exploited *in vitro* an *in vivo*.

#### I like to move it

The health benefits of physical exercise are beyond debate, but the real difficulty lies not in which fitness channel to follow on social media, but rather in adjusting the type and amount of effort in order to maximise the favourable effects and avoid damage by excessive training. Exercise indeed induces both pro- and anti-inflammatory signalling, and can have far-reaching effects on overall health and susceptibility to infection and disease [54].

Despite it being currently quite fashionable to consider gender a matter of personal choice and public debate, biological sex and the linked morphological and hormonal differences do have a significant impact on many physiological processes, notably the immune response [55].

Here, Vela et al. aimed to assess potential sex differences regarding the inflammatory response, oxidative stress levels, and muscle damage after acute resistance exercise [56].

The authors conducted the study with remarkable effort to control for as many external variables as possible, such as nutrition and hormonal cycle. They observe a stronger effect on the pro-inflammatory response and on lipid peroxidation 24 h after effort specifically in men, and advance the hypothesis that oestrogen could display a protective role against Il-6 release and oxidative stress [56].

#### Le grand mal

“To seize, to possess, to afflict” is the signification of the Ancient Greek root for epilepsy, one of the most frequent neurological disorders affecting 1–3% of the population worldwide. Even nowadays, it is still considered a sign of demonic possession in some regions, despite the fact that the “Father of Medicine”, Hippocrates himself, campaigned against the divine or hellish vision of a medical problem in the 5th century BC [57].

The disease consists in the sudden synchronous activity of cortical neurons, leading to epileptic seizures of varying intensity. Some underlying genetic factors, brain injuries, tumours, or dysregulated microRNAs have been identified to be the cause of epilepsy, but in 60% of the cases, the source is unknown [58]. Substantial progress has been made in terms of treatment, comprising anti-convulsants [59], neuromodulation [60], and dietary regulations, such as ketogenic diet [61]. Nonetheless, about a third of patients continue to experience seizures [61], leaving surgery as the sole remaining option.

Hsieh et al. conducted a retrospective study relating to the outcomes of thirty patients who underwent different types of epilepsy surgery at the Chang-Gung Memorial hospital in Taiwan [62]. They provide a detailed description of their patient classification system and the type of surgery performed. Overall, a good outcome was observed in 86.7% of cases, and the authors recommend extensive surgical removal of the epileptic foci and surrounding tissue, possibly combined with multiple subpial cortical transections, in order to maximise the chances to achieve seizure freedom.

#### Real life photoshop

Double jaw surgery, also known as bi-maxillary osteotomy, aims at correcting both aesthetic and functional skeletal issues, such as a protruding jaw, significant malocclusion, obstructive sleep apnea, or facial deformities [63].

Computer-aided design and computer-aided manufacturing (CAD/CAM), as well as the 3D-printing technology, have been proven of major usefulness in the simulation of orthognathic surgery and the custom-tailored synthesis of splints and guides, taking over from the traditional plaster cast models [64,65].

Here, Niu et al. report on their novel design of an occlusal splint for intraoperative confirmation of the soft and hard tissue results during single-splint two-jaw orthognathic surgery [66]. Notably, they added extension bars that supplied the surgeons with convenient intraoperative guidance. The authors observe a very satisfying degree of similarity between the conventional plaster dental model and the CAD/CAM surgical splints, and a good translation of the virtual plan into practical surgery. The procedure was judged convenient by the personnel and the results entirely to the satisfaction of the patients, without the requirements of secondary intervention [66].

### Brief communication

#### Cyanide and happiness

Fake teeth with hidden compartments are an evergreen favourite of espionage films and literature, just as much as the “suicide pill”, a tiny capsule filled with potassium cyanide. Both elements have actually a solid basis in real history, their combination into a “cyanide tooth” is however mostly fiction, because the pea-sized pill would have been too large for a realistic tooth. Instead, they were hidden in glasses and pens, or worn on necklaces, while the tooth compartments concealed rather microfilms.^10^

But real teeth do hold hidden treasures, in the form of dental pulp stem cells (DPSCs) [67,68]. Recently discovered by regenerative therapy enthusiasts, they are an appealing source of easily accessible adult mesenchymal stem cells with a penchant for neurogenic differentiation [69]. This does not come as a surprise, considering that DPSCs originate from the neural crest [70], nor does the fact that they remain receptive to neurotrophic factors, usually promoting neural survival and growth.

Xia et al. have previously shown that glial cell line derived neurotrophic factor (GDNF) ligands and receptors are expressed by DPSCs and involved in cell migration [71]. In this study, they complement the picture by demonstrating that human DPSCs additionally express the membrane receptor TrkB and secrete its ligand brain derived neurotrophic factor (BDNF). Moreover, the authors show that the treatment of DPSCs with exogenous BDNF and NT4/5 stimulates cell migration, but not proliferation, by triggering the MAPK pathway *via* ERK phosphorylation [72]. Altogether, this knowledge holds great potential to be applied to orchestrate the behaviour of DPSCs prior to or upon use for regenerative therapy.

#### Precision plumbing

Ischemic stroke is the result of the blockage or narrowing of an artery leading to the brain, such as the carotid artery of the neck, and causes downstream damage to the brain through hypoxia and subsequent inflammation [73]. In the majority of cases, the underlying cause is intracranial atherosclerosis (IPAD), referring to deposits of fat and cholesterol that accumulated in the arteries through a combination of underlying genetic risk factors and life-style aspects, like smoking [74]. Quick re-establishment of the blood supply is crucial following ischemic stroke. If blood-thinning medication fails, mechanical dissociation of the obstruction is required – an extremely delicate intervention given the fragility of the cerebral vascularisation, where the slightest mistake risks amplifying the damage.

In this clinical report. Lin et al. describe the successful management of ICAD in an elderly patient, more specifically a case of tight stenosis at the intradural right vertebral artery, by inserting a stent system through the proatlantal intersegmental artery (PIA) [75].

### Letters

#### Multipass, chapter II

Regular testing for SARS-CoV-2 by PCR or antigen test has become a routine in many countries, where the access to many facilities from restaurants to workplaces relies on the “corona-pass”, proof of either vaccination or a negative test from less than 72 h ago.

In a recent issue of the Biomedical Journal, You et al. compared the accuracy of the Roche cobas 6800 SARS-CoV-2 kit and the Taiwan Centres for Disease Control (CDC) protocol, finding an overall agreement of 80% between the two methods [76,77].

Poon et al. suggest in this correspondence to repeat the PCR test or analyse the sample with an alternate test in the case of late amplification in one of the viral targets [78].

#### Multipass, chapter III

In response to the correspondence by Poon et al. [78], the authors of the initial study [76,77] agree with the necessity to test for at least two independent SARS-CoV-2 targets. In addition, they point out the diversity of possible sources that can affect the accuracy of the test result, from mutations to flawed sampling, which speak in favour of re-sampling rather than re-testing the questionable sample [79].

## Conflicts of interest

The author declares no conflict of interests.

## Footnotes

1 https://www.grayflannelsuit.net/blog/celebrity-smokes-a-gallery-of-star-powered-cigarette-ads.

2 https://www.thewonderland.co.uk/blog/publicity-can-come-from-the-most-unexpected-corners/.

3 https://www.businessinsider.com/vintage-tobacco-adverts-that-would-be-illegal-today-2016-6?r=US&IR=T.

4 https://www.lemonde.fr/societe/article/2009/11/20/gainsbourg-interdit-de-fumer-par-la-ratp_1270111_3224.html.

5 https://www.who.int/fctc/text_download/en/.

6 https://www.cancer.org/cancer/lung-cancer/causes-risks-prevention/risk-factors.html.

7 https://www.bbc.com/news/health-24914473.

8 https://retractionwatch.com/2021/04/21/journal-retracts-paper-suggesting-smoking-is-linked-to-lower-covid-19-risk/.

9 https://www.ndemiccreations.com/en/22-plague-inc.

10 https://www.atlasobscura.com/articles/cyanide-tooth-is-not-real.

## References

[1] Proctor R.N. (2012). The history of the discovery of the cigarette-lung cancer link: evidentiary traditions, corporate denial, global toll. Tobac Control.

[2] Samet J.M. (2013). Tobacco smoking: the leading cause of preventable disease worldwide. Thorac Surg Clin.

[3] Kondo T., Nakano Y., Adachi S., Murohara T. (2019). Effects of tobacco smoking on cardiovascular disease. Circ J.

[4] Messner B., Bernhard D. (2014). Smoking and cardiovascular disease: mechanisms of endothelial dysfunction and early atherogenesis. Arterioscler Thromb Vasc Biol.

[5] Rabe K.F., Watz H. (2017). Chronic obstructive pulmonary disease. Lancet.

[6] Thomsen S.F., Sørensen L.T. (2010). Smoking and skin disease. Skin Ther Lett.

[7] Durazzo T.C., Mattsson N., Weiner M.W. (2014). Alzheimer's Disease Neuroimaging Initiative. Smoking and increased Alzheimer's disease risk: a review of potential mechanisms. Alzheimers Dement.

[8] Alexandridi F., Tsantila S., Pepelassi E. (2018). Smoking cessation and response to periodontal treatment. Aust Dent J.

[9] McEvoy C.T., Spindel E.R. (2017). Pulmonary effects of maternal smoking on the fetus and child: effects on lung development, respiratory morbidities, and life long lung health. Paediatr Respir Rev.

[10] Peterson L.A., Hecht S.S. (2017). Tobacco, e-cigarettes, and child health. Curr Opin Pediatr.

[11] Dinardo P., Rome E.S. (2019). Vaping: the new wave of nicotine addiction. Cleve Clin J Med.

[12] McKee M. (2014). Electronic cigarettes: peering through the smokescreen. Postgrad Med J.

[13] Almeida-da-Silva C.L.C., Dakafay H.M., O'Brien K., Montierth D., Xiao N., Ojcius D.M. (2021). Effects of electronic cigarette aerosol exposure on oral and systemic health. Biomed J.

[14] Yuan M., Cross S.J., Loughlin S.E., Leslie F.M. (2015). Nicotine and the adolescent brain. J Physiol.

[15] Benowitz N.L., Burbank A.D. (2016). Cardiovascular toxicity of nicotine: implications for electronic cigarette use. Trends Cardiovasc Med.

[16] Huang C., Shi G. (2019). Smoking and microbiome in oral, airway, gut and some systemic diseases. J Transl Med.

[17] Savin Z., Kivity S., Yonath H., Yehuda S. (2018). Smoking and the intestinal microbiome. Arch Microbiol.

[18] Winnicka L., Shenoy M.A. (2020). EVALI and the pulmonary toxicity of electronic cigarettes: a review. J Gen Intern Med.

[19] Kligerman S., Raptis C., Larsen B., Henry T.S., Caporale A., Tazelaar H. (2020). Radiologic, pathologic, clinical, and physiologic findings of electronic cigarette or vaping product use-associated lung injury (EVALI): evolving knowledge and remaining questions. Radiology.

[20] Hendlin Y.H., Vora M., Elias J., Ling P.M. (2019). Financial conflicts of interest and stance on tobacco harm reduction: a systematic review.

[21] Oakes J.M., Fuchs R.M., Gardner J.D., Lazartigues E., Yue X. (2018). Nicotine and the renin-angiotensin system. Am J Physiol Regul Integr Comp Physiol.

[22] Engin A.B., Engin E.D., Engin A. (2020). Two important controversial risk factors in SARS-CoV-2 infection: obesity and smoking. Environ Toxicol Pharmacol.

[23] Alqahtani J.S., Oyelade T., Aldhahir A.M., Alghamdi S.M., Almehmadi M., Alqahtani A.S. (2020). Prevalence, severity and mortality associated with COPD and smoking in patients with COVID-19: a rapid systematic review and meta-analysis. PloS One.

[24] Vardavas C.I., Nikitara K. (2020). COVID-19 and smoking: a systematic review of the evidence. Tob Induc Dis.

[25] Kashyap V.K., Dhasmana A., Massey A., Kotnala S., Zafar N., Jaggi M. (2020). Smoking and COVID-19: adding fuel to the flame. Int J Mol Sci.

[26] Puri N., Coomes E.A., Haghbayan H., Gunaratne K. (2020). Social media and vaccine hesitancy: new updates for the era of COVID-19 and globalized infectious diseases. Hum Vaccines Immunother.

[27] Li Q., Wu J., Nie J., Zhang L., Hao H., Liu S. (2020). The impact of mutations in SARS-CoV-2 spike on viral infectivity and antigenicity. Cell.

[28] Collier D.A., De Marco A., Ferreira I.A.T.M., Meng B., Datir R.P., Walls A.C. (2021). Sensitivity of SARS-CoV-2 B.1.1.7 to mRNA vaccine-elicited antibodies. Nature.

[29] Antony P., Vijayan R. (2021). Role of SARS-CoV-2 and ACE2 variations in COVID-19. Biomed J.

[30] Lai C.C., Ko W.C., Lee P.I., Jean S.S., Hsueh P.R. (2020). Extra-respiratory manifestations of COVID-19. Int J Antimicrob Agents.

[31] Galanopoulos M., Gkeros F., Doukatas A., Karianakis G., Pontas C., Tsoukalas N. (2020). COVID-19 pandemic: pathophysiology and manifestations from the gastrointestinal tract. World J Gastroenterol.

[32] Pola Ak, Murthy K.S., Santhekadur P.K. (2021). COVID-19 and gastrointestinal system: a brief review. Biomed J.

[33] Chen Y., Klein S.L., Garibaldi B.T., Li H., Wu C., Osevala N.M. (2021). Aging in COVID-19: vulnerability, immunity and intervention. Ageing Res Rev.

[34] Soiza R.L., Scicluna C., Thomson E.C. (2021). Efficacy and safety of COVID-19 vaccines in older people. Age Ageing.

[35] Granier C., Gey A., Roncelin S., Weiss L., Paillaud E., Tartour E. (2021). Immunotherapy in older patients with cancer. Biomed J.

[36] Dobosz P., Dzieciątkowski T. (2019). The intriguing history of cancer immunotherapy. Front Immunol.

[37] Ponting C.P., Oliver P.L., Reik W. (2009). Evolution and functions of long noncoding RNAs. Cell.

[38] Guttman M., Amit I., Garber M., French C., Lin M.F., Feldser D. (2009). Chromatin signature reveals over a thousand highly conserved large non-coding RNAs in mammals. Nature.

[39] Hanahan D., Weinberg R.A. (2011). Hallmarks of cancer: the next generation.

[40] Huang P.S., Chang C.C., Wang C.S., Lin K.H. (2021). Functional roles of non-coding RNAs regulated by thyroid hormones in liver cancer. Biomed J.

[41] Vander Heiden M.G., DeBerardinis R.J. (2017). Understanding the intersections between metabolism and cancer biology. Cell.

[42] Yang H.C., Wu Y.H., Yen W.C., Liu H.Y., Hwang T.L., Stern A. (2019). The redox role of G6PD in cell growth, cell death, and cancer. Cells.

[43] Yang H.C., Stern A., Chiu D.T.Y. (2021). G6PD: a hub for metabolic reprogramming and redox signaling in cancer. Biomed J.

[44] Boozari M., Hosseinzadeh H. (2021). Natural products for COVID-19 prevention and treatment regarding to previous coronavirus infections and novel studies. Phytother Res.

[45] Zhao Z., Li Y., Zhou L., Zhou X., Xie B., Zhang W. (2021). Prevention and treatment of COVID-19 using Traditional Chinese Medicine: a review. Phytomedicine.

[46] Tang W.F., Tsai H.P., Chang Y.H., Chang T.Y., Hsieh C.F., Lin C.Y. (2021). Perilla (*Perilla frutescens*) leaf extract inhibits SARS-CoV-2 via direct virus inactivation. Biomed J.

[47] Schoolman H.M., Bernstein L.M. (1978). Computer use in diagnosis, prognosis, and therapy. Science.

[48] Mayo R.C., Leung J. (2018). Artificial intelligence and deep learning - radiology's next frontier?. Clin Imaging.

[49] Udugama B., Kadhiresan P., Kozlowski H.N., Malekjahani A., Osborne M., Li V.Y.C. (2020). Diagnosing COVID-19: the disease and tools for detection. ACS Nano.

[50] Mohammadi F., Pourzamani H., Karimi H., Mohammadi M., Mohammadi M., Ardalan N. (2021). Artifical neural network and logistic regression modelling to characterize COVID-19 infected patients in local areas of Iran. Biomed J.

[51] Dunmire S.K., Verghese P.S., Balfour H.H. Jr (2018). Primary Epstein-Barr virus infection. J Clin Virol.

[52] Rühl J., Leung C.S., Münz C. (2020). Vaccination against the epstein-barr virus. Cell Mol Life Sci.

[53] Olotu F.A., Soliman M.E.S. (2021). Immunoinformatics prediction of potential B-cell and T-cell epitopes as effective vaccine candidates for eliciting immunogenic responses against Epstein-Barr virus. Biomed J.

[54] Paolucci E.M., Loukov D., Bowdish D.M.E., Heisz J.J. (2018). Exercise reduces depression and inflammation but intensity matters. Biol Psychol.

[55] Jaillon S., Berthenet K., Garlanda C. (2019). Sexual dimorphism in innate immunity. Clin Rev Allergy Immunol.

[56] Aragón-Vela J., Fontana L., Casuso R.A., Plaza-Díaz J., Huertas J.R. (2021). Differential inflammatory response of men and women subjected to an acute resistance exercise. Biomed J.

[57] Reynolds E.H. (2020). Epilepsy and neuroscience: evolution and interaction. Front Neuroanat.

[58] Brennan G.P., Henshall D.C. (2020). MicroRNAs as regulators of brain function and targets for treatment of epilepsy. Nat Rev Neurol.

[59] Ghosh S., Sinha J.K., Khan T., Devaraju K.S., Singh P., Vaibhav K. (2021). Pharmacological and therapeutic approaches in the treatment of epilepsy. Biomedicines.

[60] Davis P., Gaitanis J., Neuromodulation for the Treatment of Epilepsy: (2020). A review of current approaches and future directions. Clin Ther.

[61] Verrotti A., Iapadre G., Di Francesco L., Zagaroli L., Farello G. (2020). Diet in the treatment of epilepsy: what we know so far. Nutrients.

[62] Hsieh H.Y., Chang C.W., Cheng M.Y., Yan J.L., Lim S.N., Tseng W.E. (2021). Aggressive cytoreduction and multiple subpial cortical transections may obtain good surgical outcomes in refractory epilepsy with multiple epileptic foci. Biomed J.

[63] Khechoyan D.Y. (2013). Orthognathic surgery: general considerations. Semin Plast Surg.

[64] Haas O.L. Jr, Becker O.E., de Oliveira R.B. (2015). Computer-aided planning in orthognathic surgery-systematic review. Int J Oral Maxillofac Surg.

[65] Lin H.H., Lonic D., Lo L.J. (2018). 3D printing in orthognathic surgery - a literature review. J Formos Med Assoc.

[66] Lo L.J., Niu L.S., Liao C.H., Lin H.H. (2021). A novel CAD/CAM composite occlusal splint for intraoperative verification in single-splint two-jaw orthognathic surgery. Biomed J.

[67] Zeitlin B.D. (2020). Banking on teeth - stem cells and the dental office. Biomed J.

[68] Häfner S.J. (2020). Bargain with the tooth fairy – the savings accounts for dental stem cells. Biomed J.

[69] Mortada I., Mortada R., Bazzal Al M. (2018). Dental pulp stem cells and neurogenesis. Adv Exp Med Biol.

[70] Dupin E., Calloni G.W., Coelho-Aguiar J.M., Le Douarin N.M. (2018). The issue of the multipotency of the neural crest cells. Dev Biol.

[71] Xiao N., Yu W.Y., Liu D. (2018). Glial cell-derived neurotrophic factor promotes dental pulp stem cell migration. J Tissue Eng Regen Med.

[72] Xiao N., Thor D., Yu W.Y. (2021). Neurotrophins BDNF and NT4/5 accelerate dental pulp stem cell migration. Biomed J.

[73] Barthels D., Das H. (2020). Current advances in ischemic stroke research and therapies. Biochim Biophys Acta Mol Basis Dis.

[74] Flusty B., de Havenon A., Prabhakaran S., Liebeskind D.S., Yaghi S. (2020). Intracranial atherosclerosis treatment: past, present, and future. Stroke.

[75] Lin C.M., Chang C.H., Wong H.F. (2021). Management of intracranial vertebral artery stenosis with ipsilateral vertebral artery hypoplasia and contralateral vertebral artery occlusion via type 2 proatlantal intersegmental artery. Biomed J.

[76] Häfner S.J. (2021). Level up for culture models - how 3D cell culture models benefit SARS-CoV-2 research. Biomed J.

[77] You H.L., Lin M.C., Lee C.H. (2021). Comparison of the Roche cobas 6800 SARS-CoV-2 test and the Taiwan CDC protocol for the molecular diagnosis of COVID-19. Biomed J.

[78] Poon K.S., Tee N.W.S. (2021). Realistic considerations for comparison between SARS-CoV-2 molecular diagnostic assays. Biomed J.

[79] You H.L., Lee C.H. (2021). Reply to “Comparison of the Roche cobas 6800 SARS-CoV-2 test and the Taiwan CDC protocol for the molecular diagnosis if COVID-19”. Biomed J.

